# Reproductive profile of artemisinins in albino rats

**DOI:** 10.4103/0253-7613.66846

**Published:** 2010-06

**Authors:** Bhabagrahi Rath, Jyotirmoyee Jena, Satyajit Samal, Bandana Rath

**Affiliations:** Department of Pharmacology, V.S.S Medical College, Burla, Sambalpur, Orissa, India

Sir,

*Plasmodium falciparum* malaria during pregnancy poses substantial risk to the mother and the fetus. Artemisinins are frequently prescribed but their safety during pregnancy is yet to be established and there is lack of published data on this subject. With this background, the present work was aimed to investigate the teratogenic effect, if any, of the three commonly used artemisinins, namely artesunate, artemether and arteether, in albino rats.

Adult Wistar rats (180–220 g) of either sex, inbred in our departmental animal house, were used. They were maintained on standard animal diet and water ad libitum. The study was approved by the Institutional Animal Ethics Committee. The estrous cycles of female albino rats were monitored by examining the vaginal smears. Estrous-positive rats were caged with male rats. Day 0 of pregnancy was known by sperm-positive vaginal smear. Finally, 48 pregnancy-confirmed rats were selected and randomly assigned to eight groups, with six animals in each group. The test drugs, artesunate, artemether and arteether, were supplied in pure powder form by Zydus Cadila, IPCA and Themis Pharmaceuticals, respectively, on request.

The method described by Marathe and Thomas (1986) was followed.[1] Each test drug was given in two doses: one, the minimal dose, i.e. approximately 10-times the human therapeutic dose and the other, the maximum tolerated dose, determined from pilot experiments. Distilled water was used as the vehicle for artesunate whereas arachis oil served the same for artemether and arteether. The drugs/vehicles were administered continuously from the 6^th^ to the 15^th^ day of gestation (the period of organogenesis in rats) by I.P. route as follows: group I - distilled water, 0.5 cc; group II - arachis oil, 0.5 cc; group III - artesunate, 10 mg/kg; group IV - artesunate, 20 mg/kg; group V - artemether, 8 mg/kg; group VI - artemether, 16 mg/kg; group VII - arteether, 15 mg/kg; group VIII - arteether, 30 mg/kg.

The rats were lightly anesthetized with ether on the 20^th^ day of gestation and ventral laparotomy was performed. The uterine cavity was opened and the fetuses were retrieved. The number of implantations and embryonic resorption sites (failed pregnancies) were recorded. All the fetuses were carefully examined for gross external anomalies. The body weight of each fetus (live or dead) and placenta was recorded and all the fetuses were sacrificed by putting them in a CO_2_ chamber. A midline ventral incision on each fetus examined any alterations or malformations of the viscera. Finally, fetal skeletal staining was performed to detect any skeletal malformations and the crown-rump length (CRL) and tail length (TL) were measured. Data were analyzed by applying the Kruskal-Wallis one-way ANOVA followed by Dunn’s test, while fetal and placental weight were analyzed by one-way ANOVA followed by Dunnett’s test.*P* < 0.05 was considered to be statistically significant.

The artemisinins exhibited no significant effect on the number of implantations. Significant resorption (*P* < 0.05,*P* < 0.01) was revealed in all the test groups except group III when compared with their respective control groups [Table 1]. Groups IV, VI and VIII treated with the higher doses of artesunate, artemether and arteether, respectively, revealed complete resorption of the embryos. The mean birth weight of the fetuses (both live and dead) and their placental weights in the test groups were significantly (*P* < 0.01,*P* < 0.001) lower than those of the control groups. Gross appearance of the fetuses (both live and dead) of rats treated with low doses of artemisinins (Groups III, V and VII) were normal, without any external anomalies like microcephaly, micrognathia, etc. However, the fetuses of all these groups were smaller in size in comparison to their respective controls. There was no visceral anomaly in all test groups except the absence of the gall bladder, a normal phenomenon in rats.

**Table 1:** Effect of artesunate, artemether and arteether on pregnancy and fetal skeleton in albino rats

*Treatment (dose, ip)*	*No. of resorptions*	*No. of fetuses alive*	*No. of fetuses dead*	*Fetal wt. (g)*	*Placental wt. (g)*	*Crown-rump length (mm)*	*Tail length (mm)*	*Skeletal abnormalities*
Distilled water (0.5 cc)	0.5 ± 0.34	5.6 ± 0.21	-	4.72 ± 0.18	0.66 ± 0.01	35.25 ± 0.03	13.3 ± 0.05	-
Arachis oil (0.5 cc)	0.33 ± 0.21	6 ± 0.26	-	4.78 ± 0.13	0.68 ± 0.00	35.28 ± 0.02	13.35 ± 0.01	-
Artesunate (10 mg/kg)	3.17 ± 0.6	0.5 ± 0.4 (3)	1.5 ± 0.37 (9)	3.61 ± 0.04**	0.41 ± 0.00*	32.03 ± 1.02***	11.33 ± 0.00***	4 (12) shortened bones (humerus, femur, tibia, fibula)
Artesunate (20 mg/kg)	4.5 ± 0.43*	-	-	-	0.36 ± 0.00***	-	-	-
Artemether (8 mg/kg)	4.67 ± 0.33^a^	-	(1)	(3.25)	0.39 ± 0.00^b^	31.06 (1)	10.45 (1)	-
Artemether (16 mg/kg)	4.5 ± 0.56^a^	-	-	-	0.353 ± 0.00^b^	-	-	-
Arteether (15 mg/kg)	4.16 ± 0.26^a^	-	0.5 (3)	3.39 ± 0.06^a^	0.39 ± 0.00^b^	31.17 ± 0.18^b^	10.94 ± 0.07^b^	1 (3) shortened bones (humerus, femur, tibia, fibula)
Arteether (30 mg/kg)	4.83 ± 0.48^a^	-	-	-	0.35 ± 0.0^b^	-	-	-

Values are expressed as mean ± SEM, figure in parenthesis represents the actual number,*n* = 6

* *P* < 0.05,

** *P* < 0.01,

*** *P* < 0.001 for the artesunate group as compared with the respective control, Group I

a *P* < 0.01,

b *P* < 0.001 for the artemether and the arteether groups as compared with the respective control, Group II

Further, examination of the fetal skeletons revealed significantly (*P*< 0.001) short CRL and TL in rats treated with lower doses of artesunate and arteether, respectively [Table 1]. Besides, four of the 12 fetuses of Group III (artesunate 10 mg/kg) and one of the three fetuses of group VII (arteether 15 mg/kg) had short limb bones, viz. humerus, femur, tibia and fibula, than that of their control litters. Our results corroborate with those of White *et al*.,[2] who found totally resorbed litters and fetuses with bent and/or shortened bones.[2] Mesembe *et al*.[3] have also found a significant reduction in the CRL and TL in rats treated with 0.4 and 0.8 mg/kg of artesunate.[3]

In the present study, artemisinins were found to have a significant embryofetal toxicity in rats without any gross malformation. This toxicity could be attributable to inhibition of angiogenesis in the yolk sac.[4] Additionally, artemisinin-like drugs may affect pituitary hormone secretion, leading to complete resorption by a mechanism that does not operate in humans.[5] Keeping in view the wide use of artemisinins, especially in multidrug-resistant *P. falciparum* malaria in pregnant women, the prescribers should be aware of the probable teratogenic risk of these drugs in spite of their approval for clinical use.
